# Demonstration of the Early Cardiac Bioavailability of a Non-Specific Cell-Targeted Peptide Using Radionuclide-Based Imaging In Vivo

**DOI:** 10.3390/ph16060824

**Published:** 2023-05-31

**Authors:** Stephan Settelmeier, Zohreh Varasteh, Magdalena Staniszewska, Anna-Lena Beerlage, Fadi Zarrad, Wolfgang P. Fendler, Christoph Rischpler, Johannes Notni, Matthias Totzeck, Ken Herrmann, Tienush Rassaf, Ulrike B. Hendgen-Cotta

**Affiliations:** 1 Department of Cardiology and Vascular Medicine, West German Heart and Vascular Center, Medical Faculty, University Hospital Essen, University of Duisburg-Essen, 45147 Essen, Germany; 2 Department of Nuclear Medicine, Medical Faculty, University Hospital Essen, University of Duisburg-Essen, 45147 Essen, Germanywolfgang.fendler@uk-essen.de (W.P.F.);; 3 Department of Nuclear Medicine, Klinikum rechts der Isar der TUM, 81675 Munich, Germany; 4 TRIMT GmbH, 01454 Radeberg, Germany

**Keywords:** nuclear cardiology, peptide drug, PET/CT, preclinical drug development, protein–protein interactions

## Abstract

The cardiac bioavailability of peptide drugs that inhibit harmful intracellular protein–protein interactions in cardiovascular diseases remains a challenging task in drug development. This study investigates whether a non-specific cell-targeted peptide drug is available in a timely manner at its intended biological destination, the heart, using a combined stepwise nuclear molecular imaging approach. An octapeptide (heart8P) was covalently coupled with the trans-activator of transcription (TAT) protein transduction domain residues 48–59 of human immunodeficiency virus-1 (TAT-heart8P) for efficient internalization into mammalian cells. The pharmacokinetics of TAT-heart8P were evaluated in dogs and rats. The cellular internalization of TAT-heart8P-Cy(5.5) was examined on cardiomyocytes. The real-time cardiac delivery of ^68^Ga-NODAGA-TAT-heart8P was tested in mice under physiological and pathological conditions. Pharmacokinetic studies of TAT-heart8P in dogs and rats revealed a fast blood clearance, high tissue distribution, and high extraction by the liver. TAT-heart-8P-Cy(5.5) was rapidly internalized in mouse and human cardiomyocytes. Correspondingly, organ uptake of hydrophilic ^68^Ga-NODAGA-TAT-heart8P occurred rapidly after injection with an initial cardiac bioavailability already 10 min post-injection. The saturable cardiac uptake was revailed by the pre-injection of the unlabeled compound. The cardiac uptake of ^68^Ga-NODAGA-TAT-heart8P did not change in a model of cell membrane toxicity. This study provides a sequential stepwise workflow to evaluate the cardiac delivery of a hydrophilic, non-specific cell-targeting peptide. ^68^Ga-NODAGA-TAT-heart8P showed rapid accumulation in the target tissue early after injection. The implementation of PET/CT radionuclide-based imaging methodology as a means to assess effective and temporal cardiac uptake represents a useful and critical application in drug development and pharmacological research and can be extended to the evaluation of comparable drug candidates.

## 1. Introduction

Despite significant advances in pharmacotherapies, cardiovascular disease remains the leading cause of morbidity and mortality worldwide [1]. Most of the pathological processes in the cardiovascular system are associated with detrimental intracellular protein–protein interactions, and covering their large interaction surfaces of 1500–3000 Å^2^ for inhibition is challenging [2]. While the use of classical biologics is limited to cell-surface or extracellular targets [3], therapeutics with intracellular targets must gain access to cells to achieve the desired biological effects [4]. Due to their small size, small molecule drugs are able to cross the cell membrane, but covering the interface area of the interaction is more feasible for peptides because of their physiochemical properties including their large size and more flexible backbone [5]. Peptide drugs account for 5% of the global pharmaceutical market [6]. They have also received increased attention as therapeutics for cardiovascular diseases, e.g., Elamipretide targeting mitochondrial myopathy [7,8]. Most peptide drugs modulate peripheral extracellular targets due to their membrane impermeability or weak membrane permeability. Evolution in cell-penetrating [9,10] and stapled [11,12,13] peptides has greatly aided the development of peptide drugs against intracellular protein–protein interactions. It has been shown that the human immunodeficiency virus type I (HIV-1) transactivator of transcription (TAT) and its core peptide segment play an important role in promoting the cellular internalization of coupled bioactive macromolecules, such as peptides as drug molecules [14]. For optimal therapeutic use, it is also important to know the fate of the drug in the body after administration. Evaluation of the pharmacokinetics, which refers to the movement of a drug into, through, and out of the body, is a prerequisite for the approval process. Basic pharmacokinetic parameters such as absorption, distribution, metabolism, and elimination (ADME) are determined in appropriate biological fluids using plasma and are calculated from the drug plasma concentration–time curve. However, information on the temporal accumulation of the drug in the target organ remains unknown. Therefore, real-time in vivo pharmacokinetic studies in preclinical biological models play a key role in predicting organ uptake and cellular internalization in target cells. PET/CT imaging is an important and elegant tool for the in vivo visualization of peptide drug delivery to tissues [14,15,16,17,18]. It also provides information on temporal organ uptake.

In this study, the cationic TAT protein transduction domain amino acid residues 48–59 (PTD, GRKKRRQRRRPQ) of HIV-1, which serves as an intracellular delivery system [18,19], was covalently coupled to an octapeptide (TAT-heart8P) and evaluated with different sequential stepwise label-free, fluorescence-labeled, and radiolabeled approaches in various biological systems. 

## 2. Results

### 2.1. TAT-heart8P Clears from Systemic Circulation by Liver Elimination

Conventional pharmacokinetic measurements were performed in canine and rat animal model organisms. Various blood pharmacokinetic parameters of TAT-heart8P obtained from non-compartmental analysis applied to the plasma concentrations of dogs and rats are given in Table 1 and Table 2. After intravenous (i.v.) bolus administration, TAT-heart8P concentrations declined in a multi-exponential manner, with elimination half-life values of 0.5 h (male dog), 0.4 h (female dog), 0.5 h (male rats), and 0.6 h (female rats). A total of 6 h after i.v. injection, TAT-heart8P was undetectable in all animals examined. TAT-heart8P was highly extracted by the liver in both model organisms. The clearance values for dogs (CL_male_ = 3110 mL/h/kg; CL_female_ = 2960 mL/h/kg) and rats (CL_male_ = 3670 mL/h/kg; CL_female_ = 3930 mL/h/kg) were greater than the liver blood flow in a 10 kg dog (1850 mL/h/kg) or 0.25 kg rat (3310 mL/kg). The apparent volume of distribution at the steady state (V_SS)_ values (male dog: 1450 mL/kg, female dog: 1350 mL/kg; male rats: 2080 mL/kg, female rats: 2310 mL/kg)) exceeded the total body water of a 10 kg dog (604 mL/kg), indicating that TAT-heart8P is widely distributed to the tissues. 

### 2.2. TAT-heart8P-Cy(5.5) Internalizes Rapidly into Cardiomyocytes

**Figure 1:**
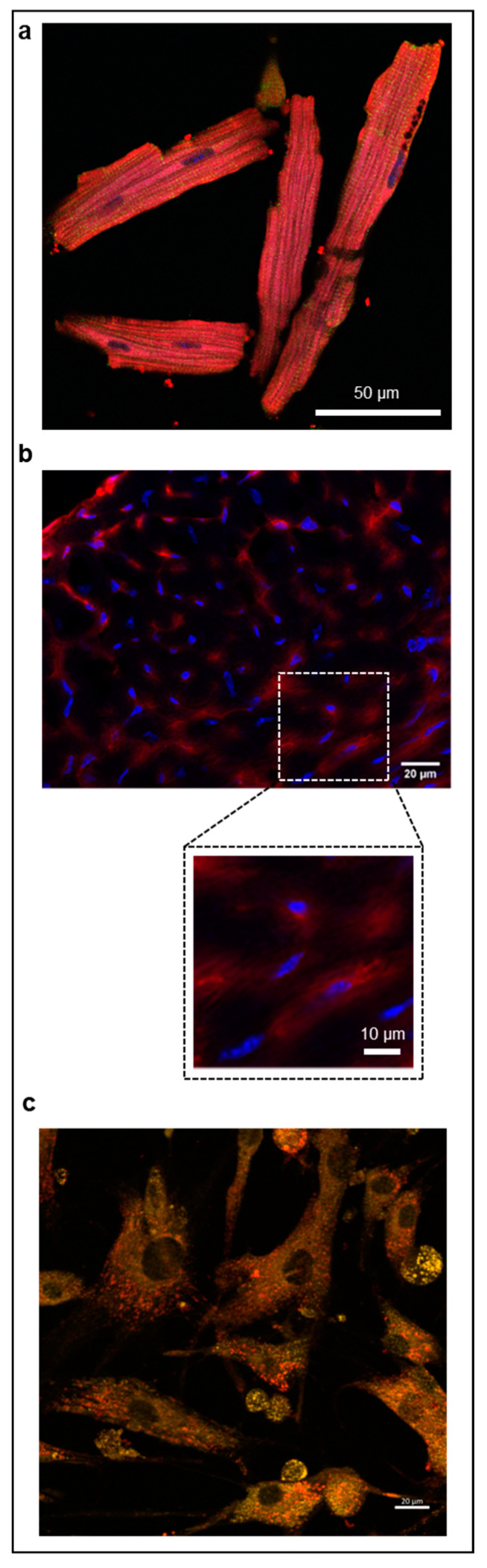
Cellular internalization of TAT-heart8P-Cy(5.5)*. (***a**) Representative confocal glycerin-immersion micrograph of an in vitro internalization of TAT-heart8P-Cy(5.5) in isolated mouse cardiomyocytes (blue: nuclei, green: troponin, red: Cy(5.5)) (magnification: 63×; scale bar 50 µm) and (**b**) ex vivo image of the in vivo uptake in left-ventricular tissue of mice showing a high internalization rate of TAT-heart8P-Cy(5.5) 10 min after injection (blue: nuclei, red: Cy(5.5)) (magnification: 63×; insert: digital 2×; scale bars 20/10 µm). (**c**) Representative confocal micrograph of an in vitro internalization of TAT-heart8P-Cy(5.5) in primary human cardiomyocytes (green: autofluorescence, red: Cy(5.5)) (magnification: 40×; scale bar 20 µm).

In vitro studies in mouse and human cardiomyocytes as well as ex vivo analysis of mouse cardiac tissues demonstrated the rapid intracellular internalization of TAT-heart8P-Cys(5.5) (Figure 1). 

### 2.3. Radiolabeling and Radiochemical Purity 

**Figure 2:**
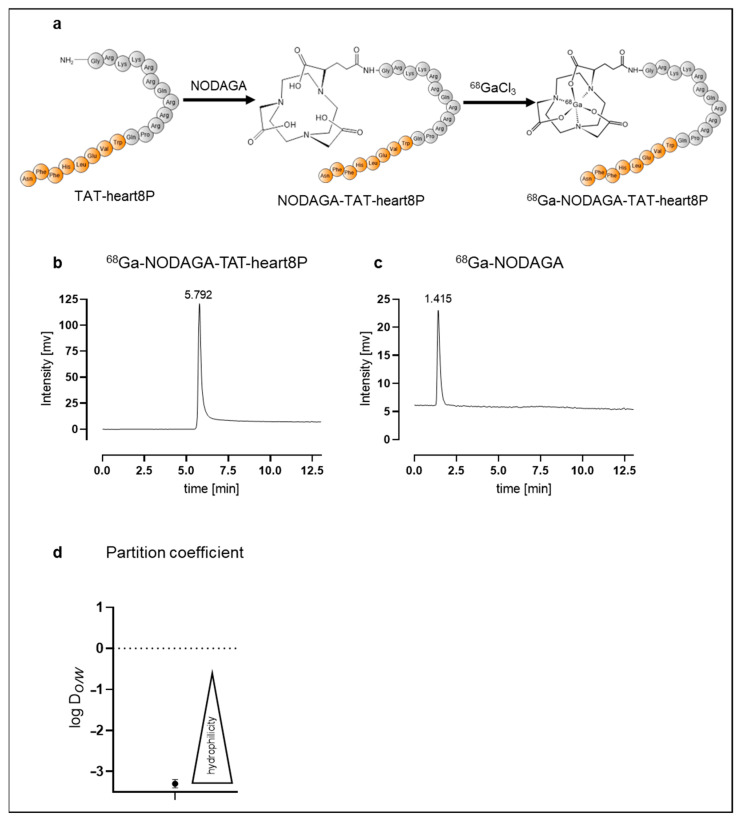
(**a**) Schematic structure of TAT-heart8P, NODAGA-TAT-heart8P, and ^68^Ga-NODAGA-TAT-heart8P. Radio-HPLC showing peaks for (**b**) ^68^Ga-NODAGA-TAT-heart8P at 5.8 min and (**c**) ^68^Ga-NODAGA at 1.4 min. Heart8P is displayed in orange. (**d**) Partition coefficient determined by octanol-water method indicates high hydrophilicity.

In order to obtain detailed information on in vivo bioavailability in addition to the calculated conventional pharmacological data, a traceable ^68^Ga-1,4,7-triazacyclononane-1-glutaric acid-4,7-acetic acid(NODAGA)-TAT-heart8P peptide conjugate was designed. The chemical structures are shown in Figure 2a. NODAGA-TAT-heart8P was labeled with ^68^Ga with an overall radiochemical yield of 95 ± 1% and radiochemical purity of >95%, before purification. The molar activity was 12 ± 3 GBq/μmol.

Using radio-HPLC, the radiochemical purity of the purified ^68^Ga-NODAGA-TAT-heart8P peptide conjugate was over 99%. The retention time of ^68^Ga-NODAGA-TAT-heart8P was 5.8 min (Figure 2b). ^68^Ga-NODAGA served as the control, with a retention time of 1.4 min (Figure 2c). The partition coefficient of log *D*_o/w_ = −3.1 ± 0.2 indicated that ^68^Ga-NODAGA-TAT-heart8P is highly hydrophilic (Figure 2d). 

### 2.4. Stability Tests: ^68^Ga-NODAGA-TAT-heart8P Is Stable for the Time Points Studied

**Figure 3:**
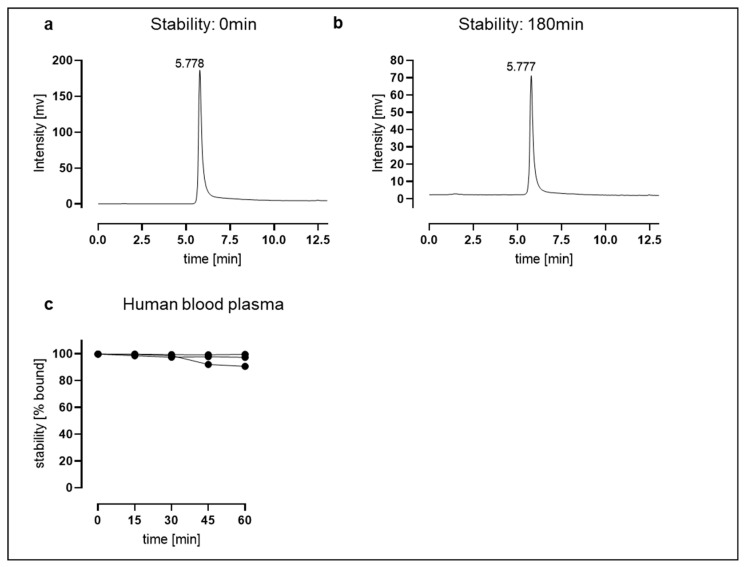
In vitro and ex vivo stability. ^68^Ga-NODAGA-TAT-heart8P was >98% stable in aqueous solution after 180 min. Radio-HPLC showing peaks for ^68^Ga-NODAGA-TAT-heart8P at (**a**) 0 min and after (**b**) 180 min. (**c**) Ex vivo stability of ^68^Ga-NODAGA-TAT-heart8P in human blood plasma of three individual donors was 96 ± 3% after 60 min of incubation at 37 °C.

The in vitro stability of ^68^Ga-NODAGA-TAT-heart8P was >98% assessed in an aqueous solution at 37 °C for 180 min (Figure 3a,b). Stability was also assessed in human blood plasma at 37 °C for up to 60 min with the aim of investigating the release of free ^68^Ga and the trans-chelation of ^68^Ga to serum proteins. The ^68^Ga-NODAGA-TAT-heart8P peptide conjugate was stable after 15 min (99.3 ± 0.6%) and decreased slightly to 96 ± 4% after 60 min of incubation in human plasma at physiological temperature. The retention time was 5.4 min after 15 min of incubation of the conjugate in human blood plasma (Figure 3c and Figure S1). Within the cardiac tissue, the compound is subject to multiple degradation processes. Therefore, we exposed the ^68^Ga-NODAGA-TAT-heart8P peptide conjugate to murine whole heart tissue lysate to simulate in-tissue metabolization. While the initial stability after 15 min of incubation at 37 °C was 96 ± 3%, it decreased to 42 ± 3% after 60 min (Figure S2a), due to peptidase activity rather than de-chelation (Figure S2b). To test the degradability of the product, we used proteinase K as a positive control. Incubation with proteinase K decreased the stability of ^68^Ga-NODAGA-TAT-heart8P to 73 ± 14% and 46 ± 18% of the intact tracer after 15 and 60 min, respectively (Figure S3). In vitro stability studies showed that ^68^Ga-NODAGA-TAT-heart8P was stable for the time period studied.

### 2.5. Cardiac Bioavailability of ^68^Ga-NODAGA-TAT-heart8P

**Figure 4:**
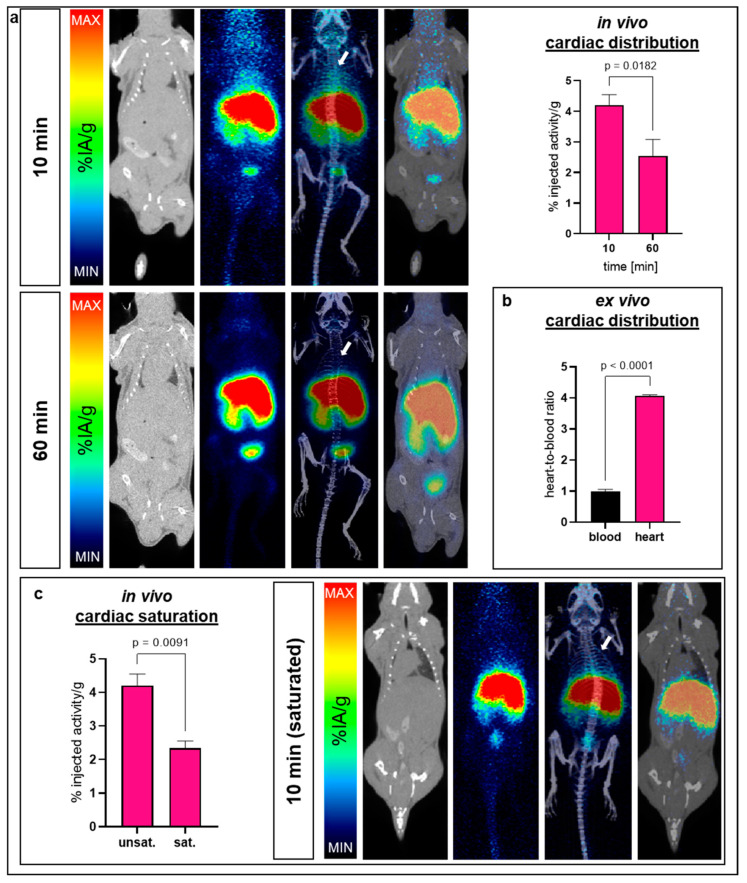
Cardiac bioavailability of ^68^Ga-NODAGA-TAT-heart8P. (**a**) Representative ^68^Ga-NODAGA-TAT-heart8P images (CT, PET, fused PET/CT, maximum intensity projection (MIP)) of healthy C57BL/6J mice 10 and 60 min after injection. Average cardiac distribution (%IA/g) is displayed on the right. Cardiac accumulation significantly decreased after 60 min (*p* = 0.0182). White arrows indicate the anatomical position of the heart. MIN/MAX (10 min) = 0/50 %IA/g; MIN/MAX (60 min) = 0/30 %IA/g. *n* = 3–9. Data are presented as the mean ± SEM. (**b**) Ex vivo heart-to-blood ratio proofed cardiac tissue accumulation of ^68^Ga-NODAGA-TAT-heart8P 10 min after i.v. injection determined by γ-counter measurements. Significantly increased heart-to-blood ratio indicates cardiac tissue accumulation of blood-free myocardial tissue (*p* < 0.0001) (*n* = 3). Data are presented as the mean ± SEM. (**c**) Saturability after the injection of unlabelled NODAGA-TAT-heart8P. Accumulation after 10 min is significantly reduced by previous saturation (*p* = 0.0091) (compare unsaturated data in **a**). Representative (CT, PET, fused PET/CT, MIP) images of saturated experiments. MIN/MAX (10 min) = 0/50 %IA/g. Data are presented as the mean ± SEM (*n* = 3 (pre-injected with non-labeled compound) to 9 (injected only with radiolabeled compound)). Adjustment of MIN/MAX for comparability because of declined activity.

Evaluated in C57BL/6J mice, the cardiac uptake of ^68^Ga-NODAGA-TAT-heart8P was 4.3 ± 0.7% at 10 min after injection and decreased further to 2.5 ± 0.6% at 60 min (Figure 4a). Gamma counter-based heart-to-blood ratio data of blood-free heart tissue evidenced a significant uptake (ratio 4:1) in cardiac tissue as early as 10 min after i.v. injection (Figure 4b). The pre-injection of 100 µg of unlabeled NODAGA-TAT-heart8P diminished the cardiac uptake of ^68^Ga-NODAGA-TAT-heart8P significantly (2.3 ± 0.4% 10 min post-injection, *p* = 0.01), indicating saturable uptake (Figure 4c). Gamma counter measurements confirmed the decreased tissue accumulation determined by PET/CT 60 min after i.v. administration (Figure S4).

### 2.6. Further Tissue Distribution: ^68^Ga-NODAGA-TAT-heart8P Rapidly Eliminated from Systemic Circulation by Liver Uptake

**Figure 5:**
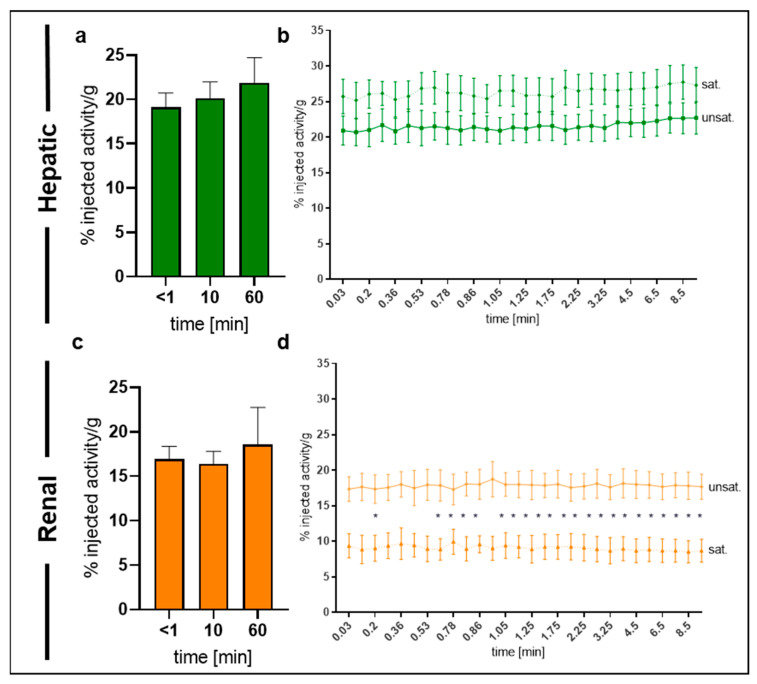
Tissue distribution of ^68^Ga-NODAGA-TAT-heart8P. (**a**) Overall nearly constant liver uptake over time, while (**b**) only slight facilitation can be observed in saturation experiments. (**c**) Constant kidney uptake over time, while (**d**) renal uptake is significantly reduced in saturation experiments. Representative MIP image of unsaturated experiment indicates excretion modalities. *N* = 3 (preinjected with non-labeled compound) to 6 (injected only with radiolabeled compound). Data are presented as the mean ± SEM. Ordinary one-way (**a**,**c**)/two-way (**b**,**d**) ANOVA with Bonferroni correction. * *p* ≤ 0.05.

Overall, ^68^Ga-NODAGA-TAT-heart8P showed a major uptake in the liver within the first 10 min after i.v. administration, followed by the kidneys. The hepatic and renal uptake of ^68^Ga-NODAGA-TAT-heart8P was 22 ± 3% and 19 ± 4%, respectively, 60 min after injection (Figure 5a,c). In saturation experiments, we observed a slight but not significant enhancement of liver uptake (Figure 5b). However, the renal uptake was significantly reduced (*p* ≤ 0.05) by the pre-injection of unlabeled TAT-NODAGA-heart8P, indicating a saturable renal uptake mechanism (Figure 5d).

### 2.7. Tissue Distribution in the Pathological Mouse Model

**Figure 6:**
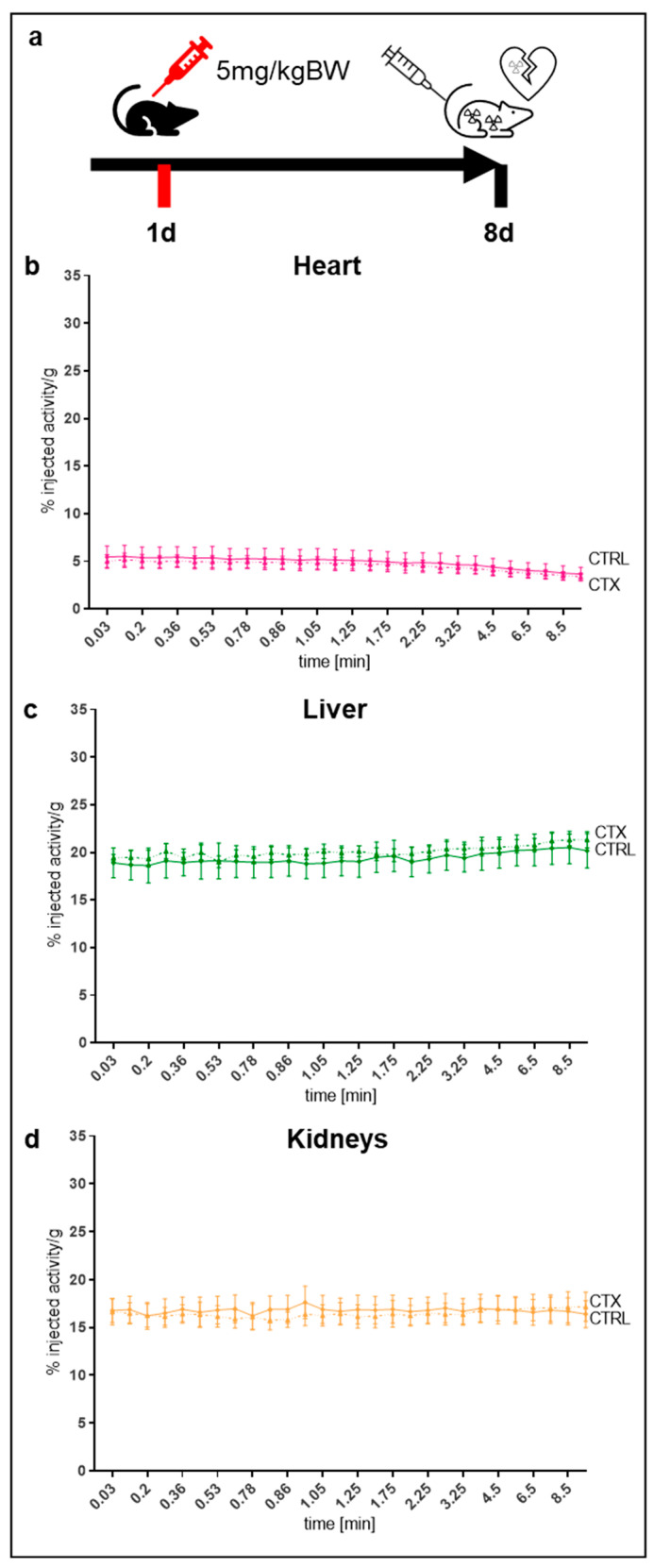
(**a**) Injection scheme of Doxorubicin (DOX) treatment. (**b**) Cardiac, (**c**) liver, and (**d**) renal uptake of ^68^Ga-NODAGA-TAT-heart8P in the first 10 min after i.v. application in DOX-treated mice (CTX) displayed in % injected activity/gram. Data are displayed as the mean ± SEM (*n* = 5–6). Ordinary two-way ANOVA with Bonferroni correction.

To evaluate the uptake of ^68^Ga-NODAGA-TAT-heart8P in cell membrane toxicity, we used Doxorubicin (DOX) to induce membrane toxicity damage (Figure 6a) and evaluated the cardiac bioavailability and tissue distribution of ^68^Ga-NODAGA-TAT-heart8P. The cardiac uptake within the first 10 min after i.v. injection was not significantly different between DOX-treated and non-treated control mice (3.8 ± 0.2% vs. 4.0 ± 0.8%, *p* = 0.7). Overall, the tracer uptake in the heart, liver, and kidney was comparable to that of the control group in the pathological mouse model.

## 3. Discussion

A newly developed peptide with an intracellular delivery mechanism was evaluated using three (i) label-free, (ii) fluorescence-labeled, and (iii) radio-labeled approaches. Each approach provided valuable information on the temporo-spatial bioavailability of the peptide drug candidate. Conventional pharmacokinetic studies demonstrated rapid clearance from the systemic circulation with predicted high liver uptake. The Cy(5.5)-coupled approach proved rapid cell internalization in mouse and human cardiomyocytes. The ^68^Ga-labeled variant showed early cardiac bioavailability after i.v. injection and high liver uptake, consistent with calculated data from conventional pharmacokinetics. In particular, the ^68^Ga-coupled approach allows for longitudinal real-time data acquisition in the same organism.

Peptide drugs that inhibit deleterious intracellular protein–protein interactions are attracting increasing attention in the treatment of cardiovascular diseases, as they have the potential for greater stability and higher cellular internalization than vector-based delivery options [20,21]. Despite the extensive preclinical discovery of important molecular pathways and subsequent pharmacotherapeutic development, a remarkable number of drug candidates targeting intracellular signaling in cardiovascular diseases failed in clinical translation [22]. The essential question is whether the drug candidate reaches its target organ, the heart, in a timely manner. Once a drug enters the systemic circulation, it is distributed to the body’s tissue to some extent. Conventional pharmacokinetics of a drug candidate, obtained from the integral of the area under the plasma concentration curve, provide important basic information about the time course of its absorption, bioavailability, distribution, metabolism, and excretion. The volume of distribution provides a reference for the plasma concentration expected at a given dose but does not provide information on the specific organ pattern of uptake. 

The absence of cardiac tissue-specific surface receptor proteins, which limits cardiotropic drug delivery using traditional systems such as antibodies, further complicates cardiac-specific drug development. For this reason, non-receptor-based cardiac therapies using cell-penetrating peptides represent a promising approach, albeit with non-specific tissue distribution (29). However, side effects due to the lack of targeted tissue delivery, non-specific tissue distribution, and a low effective target organ concentration at the maximum tolerated dose should be investigated. 

In particular, hydrophobic peptides with cell-penetrating properties inherit the disadvantage of transduction to a wide variety of tissues, which limits their in vivo adoption [23]. To overcome this downside, TAT-heart8P was designed to be hydrophilic, and its cellular uptake was investigated in several species derived from the International Conference on Harmonization (ICH) guidelines. Ex vivo analyses of fluorescence-labeled peptides (23) allow for a high spatial resolution down to the cellular level but lack data on the temporal in vivo behavior of the designed drugs. Therefore, radionuclide-based approaches are valuable tools for the real-time in vivo monitoring of the pharmacokinetics and bioavailability of a potential drug [24,25]. To extend the information derived from basic pharmacokinetic data, TAT-heart8P was labeled with ^68^Ga. The bifunctional NODAGA chelator, which contains a metal-binding motif that allows for radiolabeling with ^68^Ga [15,16,26,27], was selected for this purpose.

Rapid clearance from the circulation confirmed no residual binding to serum proteins such as albumin [28]. Liver and tissue distribution data from conventional plasma pharmacokinetic studies were confirmed by ^68^Ga-NODAGA-TAT-heart8P uptake data.

The rapid cardiac accumulation of ^68^Ga-NODAGA-TAT-heart8P was confirmed by PET/CT scans. Due to spillover artifacts resulting from high liver accumulation, especially to differentiate cardiac tissue uptake, ex vivo measurements were performed. High cardiac tissue-to-blood ratios were calculated as early as 10 min post-injection of ^68^Ga-NODAGA-TAT-heart8P. Pre-injection with a non-labeled compound revealed saturable ^68^Ga-NODAGA-TAT-heart8P accumulation in cardiac tissue. 

Depending on their size, many peptides and peptide drugs are mainly renally cleared from the systemic blood circulation after intravenous injection. Peptides with a molecular weight below 25 kDa are susceptible to glomerular filtration without tubular reabsorption [29]. As expected [15], a high renal uptake and urinary activity were observed after the intravenous administration of a small hydrophilic compound with a molecular weight of approximately 3 kDa. Interestingly, the uptake was partially saturable, indicating not only glomerular filtration but also a cellular uptake mechanism. The liver uptake of ^68^Ga-NODAGA-TAT-heart8P was the highest of all organs examined and was high shortly after intravenous injection, indicating a relevant direct uptake mechanism and rapid internalization of ^68^Ga-NODAGA-TAT-heart8P due to its intracellular delivery system. The liver has an extensive sinusoidal network with a large endothelial surface. Considering the high tissue perfusion of the liver, the non-specific uptake and the fast cell-penetrating properties of TAT-heart8P resulted in a high liver uptake of ^68^Ga-NODAGA-TAT-heart8P. The gastrointestinal tract PET signal indicated hepatic metabolism of the compound, followed by biliary excretion.

Peptides using TAT-sequences as the cell-penetrating moiety have been reported to cross the intact blood–brain-barrier [30,31]. However, results vary between different studies, depending on the application mechanism and the animal model used [32]. In this study, the brain uptake of ^68^Ga-NODAGA-TAT-heart8P was minimal, with a negligible brain-to-blood ratio.

To further investigate the uptake of ^68^Ga-NODAGA-TAT-heart8P in a pathological model, we used DOX-induced cell membrane toxicity as a representative experimental approach. DOX-induced cardiotoxicity is a major cause of heart failure in cancer survivors [33]. Multiple mechanisms are involved in DOX-induced heart failure [34]. In addition to overt cardiotoxicity, DOX induces metabolic changes and affects cell membrane properties [35,36], which may interfere with peptide drugs designed for, e.g., cardioprotection [37]. Cardiac uptake within the first 10 min after the i.v. injection of ^68^Ga-NODAGA-TAT-heart8P did not differ significantly between DOX-treated and non-treated mice. 

It should be noted that some limitations apply to our study. In a small-animal mouse model, the PET-based measurement of the blood pool using cardiac chamber VOIs, as in humans, is limited. Therefore, blood pool activity was integrated into the VOI measurements because blood activity did not differ between the groups. To overcome this downside, ex vivo measurements of the tissue-to-blood ratio are desirable. Liver spillover artifacts may limit information on spatial myocardial distribution in the small animal model, especially in the inferior wall. Therefore, ex vivo analyses using, e.g., γ-counter based measurements were mandatory. This study did not examine metabolites, e.g., in urine. Possible toxic side effects and immunological reactions could not be addressed with the approaches used with small tracer amounts. Therefore, comprehensive metabolite studies should be performed prior to the clinical application of the peptide.

The radionuclide labeling approach presented here can be applied to all sequences and structures that are amenable to ester binding with the NODAGA chelator, taking into account their inherent polarity. If chromatographic detection of the target substance is not possible, fluorescence- and radionuclide-coupled studies allow for both ex vivo and in vivo investigations. In addition to TAT, there are several peptides with a membrane-penetrating function that can transport macromolecules into cells via energy-independent pathways [38,39]. Furthermore, radionuclide-based imaging techniques are available for the evaluation of liposomal drug delivery systems [40].

Due to the lack of cardiac-specific cell surface receptors, cell penetrating and internalizing drugs represent an essential component of future cardiovascular pharmaceuticals. Thus, the approach demonstrated here will enable the evaluation of novel pharmaceuticals on their way to regulatory approval by drug development agencies.

## 4. Materials and Methods

### 4.1. Material

All commercially obtained chemicals were of analytic grade and used without further purification. NODAGA-Chelator was purchased from Chematech (Dijon, France). For further information on materials, refer to the corresponding section.

### 4.2. Peptide Synthesis 

The cell-penetrating peptide (TAT-heart8P) composed of the HIV-1 TAT PTD amino acid residues 48-59 (GRKKRRQRRRPQ) [18,19,41] covalently coupled to eight amino acid residues WVELHFFN octapeptide exhibiting a random coil conformation (=TAT-heart8P) was synthesized in a solid phase resin-based methodology (JPT Peptide Technologies, Berlin, Germany). TAT-heart8P was conjugated with a fluorophore Cys(Cy5.5.) (=TAT-heart8P-Cys(Cy5.5)) or with an NODAGA-Chelator (=NODAGA-TAT-heart8P) (JPT Peptide Technologies, Berlin, Germany) (Table 3). The peptides were purified by high-pressure liquid chromatography (HPLC) in a linear gradient in 6 min with a flow of 1 mL/min on a C18 RP-HPLC column while recording the purity at 220 nm. The peptide purity and molecular weight were verified by mass spectrometry (JPT Peptide Technologies, Berlin, Germany). 

### 4.3. Pharmacokinetics of TAT-heart8P in Dogs and Rats

The experiments were performed by Labcorp Drug Development (North Yorkshire, UK) in accordance with the European Directive 2010/63/EU, the requirements of the Animals Act 1986, and the local ethical review. TAT-heart8P was formulated as a solution in water for injection (1 mg/mL). Formulations were stored at room temperature in a sealed container protected from light. The metabolic stability, elimination half-life, tissue distribution, and clearance of TAT-heart8P (pre-dose and 5, 30, 60, and 120 min and 6 and 24 h post-dose) were evaluated in a 31-week-old male and a 32-week-old female beagle (Envigo Global Services, Cumberland, VA, USA) as well as in 12-week-old male and female WI[Han] rats (*n* = 6, Charles River Laboratories, Margate, UK). Doses of 6 mg/kg and 12 mg/kg were administrated in dogs and rats, respectively. The animals were injected intravenously through a lateral tail vein. Samples were taken from the jugular vein into K_2_EDTA. Each blood sample was gently inverted by hand (8–10 times) and then placed on crushed wet ice until centrifugation within 15 min of collection at 2300*× g* for 10 min at 4 °C. The resultant plasma was separated and immediately stabilized with an equal volume of water (120 µL each). The concentration of the intact peptide in blood was analyzed using liquid chromatography, followed by tandem mass spectrometric detection (LC-MS/MS), and the elimination half-life (terminal phase, t_1/2_), volume of distribution at the steady state (V_ss_) and Clearance (CL) were calculated from the peptide dose, plasma peptide concentration, and area under the concentration–time curve (AUC).

### 4.4. Cellular Internalization in Adult Mouse and Human Cardiomyocytes

All animal procedures were performed in accordance with institutional guidelines and with approval from the local ethics committee in compliance with the European Convention for the Protection of Vertebrate Animals Used for Experimental and other Scientific Purposes (Directive 2010/63/EU, 81-02.04.2019A369). Cardiomyocytes were isolated from 12-week-old male C57BL/6J mice using a Langendorff-free method, as previously described [42]. Briefly, the mice were sacrificed by cervical dislocation. The thorax was rapidly opened, and the inferior vena cava was transected, followed by the perfusion of the EDTA buffer into the apex of the right ventricle using an automated perfusor with a flow rate of 2.95 mL/min (Ismatech VWR, Darmstadt, Germany). Later, the aorta was clamped, and the heart was harvested. The heart was then perfused via the injection of EDTA buffer for 6 min and prewarmed (37 °C) perfusion buffer (NaCl 130 mM, KCl 5 mM, NaH_2_PO_4_ 0.5 mM, HEPES 10 mM, Glucose 10 mM, Taurine 10 mM, MgCl_2_ 1 mM) for 2 min. Enzymatic digestion was achieved by perfusion with warm collagenase buffer (4200 U collagenase type + 5200 U collagenase type 4 + 4 mg protease XIV in 80 mL perfusion buffer solution) for 20 min. Afterwards, the clamp was removed and the heart was manually dissociated. Stop buffer (perfusion buffer + 5% fetal bovine serum) was added, and cells were passed through a 100 μm strainer (Corning Inc, Corning, NY, USA) into a 50 mL tube. The cardiomyocytes were then left to gravity for 10 min. The supernatant containing non-cardiomyocytes and debris was removed; the cardiomyocyte fraction in the pellet was then subjected to gravity settling in three steps with the reintroduction of calcium (0 mM, 1.02 mM, 1.36 mM). The cells were then resuspended in prewarmed medium (M199 + 5% FBS, 50 µg/mL Gentamicin, 50 ng/mL Amphoterecin B) and plated in laminin-coated chamber slides with a concentration of 2000 cells (200 µL)/well (Ibidi, Martinsried, Germany). 

For internalization experiments, the cells were fixed with paraformaldehyde (4%, Morphisto, Offenbach, Germany) and incubated with 7 µg of TAT-heart-8P-Cys(Cy5.5) for 2 h. Troponin counterstaining was performed using Invitrogen 1H11L19 (1:100) antibody with secondary antibody Abcam ab150077 goat-anti-rabbit Alexa Fluor 488 (1:200) and DAPI (1:4000). The uptake was evaluated by confocal microscopy (Leica SP8, Wetzlar, Germany). Primary human cardiomyocytes (HCM) were purchased from Promocell GmbH, Heidelberg, Germany and cultured according to the manufacturer’s guidelines. HCM were fixed with 4% paraformaldehyde and incubated with 2.2 µg TAT-heart-8P-Cys(Cy5.5) for 6 h. The uptake was evaluated by confocal microscopy.

For ex vivo cellular uptake analysis, C57BL6/J mice were injected with 16 nmol (=44 µg) TAT-heart8P-Cys(Cy5.5) in the left ventricular cavity. Mice were killed by cervical dislocation 10 min after injection. The thorax was rapidly opened, and the inferior vena cava was transected, followed by perfusion with NaCl. The heart was then extracted and transferred to the Tissue-Tek Optimum Cutting Temperature (O.C.T). compound (Sakura Finetek, Alphen, The Netherlands), frozen to –80 °C, and cut into 8 µm sections. After equilibration to room temperature, the sections were fixed with 4% paraformaldehyde, washed with TBS-T, permeabilized with TBS-T-X (0.5%), blocked with NGS (10%), and stained with DAPI.

### 4.5. Radiolabeling

A ^68^Ge/^68^Ga generator was eluted with 0.1 M aqueous HCl, and a 1 mL fraction containing the highest activity (∼300−400 MBq) was transferred into a reactor vial containing 50 µg (50 µL in water) of NODAGA-TAT-heart8P in 150 μL of 2 M sodium acetate solution to adjust the pH of the reaction mixture to 3.5. The mixture was heated at 95 °C for 5 min. The reaction mixture was then purified by C18 Cartridge. Briefly, the reaction mixture was diluted with 10 mL water and transferred to a preconditioned (with 1 mL EtOH and 10 mL Water) C18 Cartridge. The cartridge was rinsed with water and dried with air. The product was eluted with 200 µL EtOH. The radiochemical purity (RCP) of the tracer was determined by radio HPLC and radio-instant thin layer chromatography (radio-ITLC) before and after purification, as described earlier [43]. 

### 4.6. Lipophilicity

For the determination of the partition coefficient via the measurement of the distribution of radioactivity in an organic (*n*-octanol, Merck, Darmstadt, Germany), an aqueous phase (PBS) and ^68^Ga-NODAGA-TAT-heart8P peptide conjugate (100 µL, ~0.5–1 MBq) were mixed (1:1), vortexed for 3 min, and centrifuged (6000× *g*, Biofuge 15, Heraeus Sepatech, Osterode, Germany) for 5 min for the clear separation of the organic and inorganic layer. Afterwards, 100 µL of organic and inorganic layers was measured using a γ-counter (Perkin-Elmer Gamma Counter 2480 Wizard, Waltham, MA, USA). Then, the octanol-water partition coefficient log D*_o/w_* was calculated. The experiment was repeated three times with three replicates each.

### 4.7. Stability (In Vitro and Ex Vivo)

For the investigation of the in vitro plasma stability of the radiolabeled peptide, 15 mL of human venous blood (*n* = 3) was heparinized and centrifuged at 1000× *g* for 10 min for plasma separation. Informed consent was obtained from all subjects involved. ^68^Ga-NODAGA-TAT-heart8P was incubated with human plasma (1:4 ratio) for 15, 30, 45, and 60 min at 37 °C on a horizontal shaker at 300 rpm. A 20 µL fraction was applied for radio-HPLC analysis at each time point. Radio-HPLC was conducted on an Agilent 1220 Infinity II LC (Santa Clara, CA, USA) with a Chromolith Performance RP-18 100 × 4.6 mm monolithic HPLC column (Merck, Darmstadt, Germany) equipped with a radio detector (HERM LB 500, Berthold Technologies, Bad Wildbad, Germany), using Acetonitrile/water with 0.1% trifluoroacetic acid (VWR, Darmstadt, Germany/Sigma-Aldrich, St. Louis, MO, USA) as the solvent. The enzymatic degradation of ^68^Ga-NODAGA-TAT-heart8P was further assessed in C57BL/6J mouse hearts. Briefly, hearts were isolated, perfused with water, and lysed in phosphate-buffered saline (PBS). Whole tissue lysate was generated using mechanical tissue destruction in RIPA buffer (Sigma-Aldrich). Whole heart tissue lysates were then incubated with ^68^Ga-NODAGA-TAT-heart8P (1:3 ratio) for 15, 30, 45, and 60 min at 37 °C on a horizontal shaker at 300 rpm. A 20 µL fraction of each time-point was used for radio-HPLC analysis. For positive control samples, 180 µL of ^68^Ga-NODAGA-TAT-heart8P was incubated with 20 µL proteinase K (Qiagen, Düsseldorf, Germany). 

### 4.8. Bioavailability Studies in Mice: PET/CT-Imaging and Tissue Distribution

All animal procedures were performed in accordance with institutional guidelines and with approval from the local ethics committee in compliance with the European Convention for the Protection of Vertebrate Animals Used for Experimental and other Scientific Purposes (Directive 2010/63/EU, 81-02.04.2019A369). Male C57BL/6J mice (12 ± 4 weeks) were purchased from Janvier Labs (Le Genest-Saint-Isle, France). The mice were kept under a 12 h light and dark cycle with unlimited access to water and food. For PET/CT imaging, the mice were anesthetized with 2–3% isoflurane and injected with 10 MBq of ^68^Ga-NODAGA-TAT-heart8P via the tail vein. A group of animals was preinjected with 100 µg unlabeled NODAGA-TAT-heart8P 10 min before the administration of ^68^Ga-NODAGA-TAT-heart8P for saturation experiments. Dynamic PET images were acquired immediately after the injection of ^68^Ga-NODAGA-TAT-heart8P for 10 min, and static images were acquired 1 h post-injection for 15 min using a benchtop small animal PET and a self-shielded computed tomography scanner (β-CUBE and X-CUBE, Molecubes, Gent, Belgium). The mice were scanned on temperature-controlled beds with continuous monitoring of the breathing. Images were reconstructed using an iterative reconstruction algorithm (ISRA, 30 iterations), with attenuation correction of the corresponding CT image, as described previously [44]. PET data were reconstructed into a 192·192 transverse matrix, producing a 400 μm isometric voxel size. Volumes of interest (VOIs) were defined as spheres with a diameter of 2.5 mm (liver, kidney, brain) and a whole organ for the heart. Tissue uptake was calculated as the mean percentage of injected activity per gram of tissue (% IA/g) using PMOD software (version 4.1, PMOD Technologies Ltd., Zürich, Switzerland). Cell membrane damage caused by drug-related cardiotoxicity influences the biochemistry and biometabolism of the heart [45] and may therefore influence the cardiac bioavailability of cell-penetrating oligopeptides. For cardiac damage studies, 5 mg/kg Doxorubicin (DOX) [46,47] (Medac GmbH, Wedel, Germany) was injected intraperitoneally 7 days before PET/CT imaging. Following PET/CT scans, organs were collected, and tissue radioactivity concentrations were measured on an automated gamma counter (Perkin Elmer, Waltham, MA, USA). 

### 4.9. Statistical Analyses

Data were analyzed using Prism software (version 9.2.0, GraphPad software, San Diego, CA, USA). Data are presented as the mean ± standard deviation (SD) or ±standard error of the mean (SEM), as indicated. The data incorporate the decay-correction for ^68^Ga with a half-life of *t*_½_ = 68 min. A Mann–WhitneyU test or ordinary one-way or two-way ANOVA test with the Bonferroni multiple comparisons test were used to calculate the statistical significance depending on the experimental design, as indicated. *p*-values ≤ 0.05 were considered statistically significant: * denotes *p* ≤ 0.05; ** denotes *p* ≤ 0.01; **** denotes *p* ≤ 0.0001.

## 5. Conclusions

This study demonstrates that a hydrophilic non-specific cell-targeted peptide, consisting of an octapeptide bound to the cell-penetrating TAT-PTD, accumulates in cardiac tissue early after injection. An integrated approach using sequential mutually complementary label-free, fluorescence-labeled, and radiolabeled approaches ensures comprehensive data collection. The implementation of PET/CT allows for the assessment of the real-time distribution of the radiolabeled peptide drug in vivo, which, together, represent a useful and critical application in drug development and pharmacological research, encouraging further clinical translation. As the development of future novel targeted cardiovascular drugs will require intracellular effectiveness, this approach can be adapted and transferred to other suitable drug delivery systems to support the research and development process. 

## 6. Patents

T.R. and U.B.H.-C. are co-founders and shareholders of Bimyo GmbH. A patent application (US63/276,028) and international publication (WO2020/229362A1) have been filed for the use of TAT-heart8P (T.R. and U.B.-H-C.: inventors and applicants; Bimyo GmbH: applicant).

## Figures and Tables

**Table 1:** Non-compartmental pharmacokinetics in dogs. CL: clearance; VSS: distribution at steady state; AUC: area under the curve.

Sex	Number	Dose Level (mg/kg)	Time after Dose (h) Concentration (ng/mL)						
			0	0.083	0.5	1	2	6	24
Male	1	6	<10.0	3590	790	455	102	<10.0	<10.0
Female	1	6	<10.0	5020	1000	506	92.1	<10.0	<10.0
**Sex**	**Number**	**Dose level** **(mg/kg)**	**t_1/2_** **(h)**	**CL (mL/h/kg)**		**V_SS_ (mL/kg)**		**AUC_0-inf_** (h*ng/mL)	
Male	1	6	0.501	3110		1450		1930	
Female	1	6	0.432	2960		1350		2030	

**Table 2:** Non-compartmental pharmacokinetics in rats. CL: clearance; VSS: distribution at steady state; AUC: area under the curve.

Sex	Number	Dose Level (mg/kg)	Time after Dose (h) Concentration (ng/mL)						
			0	0.083	0.5	1	2	6	24
Male	3	12	<10.0	5460	1500	816	237	<10.0	<10.0
Female	3	12	<10.0	5020	1230	883	218	<10.0	<10.0
**Sex**	**Number**	**Dose level** **(mg/kg)**	**t_1/2_** **(h)**	**CL (mL/h/kg)**		**V_SS_ (mL/kg)**		**AUC_0-inf_** (h*ng/mL)	
Male	3	12	0.564	3670		2080		3080	
Female	3	12	0.584	3930		2310		2870	

**Table 3:** Chemical structure and properties of TAT-heart8P and its conjugated derivates.

Peptide Properties	
TAT-heart8P	Ac-GRKKRRQRRRPQWVELHFFN-NH2
Chemical Formula	C122H195N47O26
Molecular Weight	2736.3 g/mol
Extinction Coefficient	5690 M^−1^ cm^−1^
Iso-electric point, theoretical	pH 12.81
Net charge at pH7	7.1
Average hydrophilicity	0.77
Specification (HPLC)	>90%
Purity (MS)	95.2%
Fluorophore	Ac-GRKKRRQRRRPQWVELHFFN-Cy(5.5)-NH2
Molecular Weight	3544.68 g/mol
Specification (HPLC)	>80%
Purity (MS)	89.7%
N-Term	NODAGA(GRKKRRQRRRPQWVELHFFN-NH2)
Molecular Weight	3050.50 g/mol
Specification (HPLC)	>90%
Purity (MS)	95.8%

## Data Availability

Data is contained within the article and Supplementary Material.

## Supplementary Materials

The following supporting information can be downloaded at: https://www.mdpi.com/article/10.3390/ph16060824/s1, Figure S1: Radio-HPLC graph of incubation of compound in human blood plasma. Figure S2: Stability and Radio-HPLC graph of compound in murine whole herat tissue lysate, Figure S3: Stability and Radio-HPLC graph of incubation of compound in Proteinase K. Figure S4: Tissue accumulation after 10 and 60 min.

## References

[1] Roth G.A., Mensah G.A., Johnson C.O., Addolorato G., Ammirati E., Baddour L.M., Barengo N.C., Beaton A.Z., Benjamin E.J., Benziger C.P. (2020). Global Burden of Cardiovascular Diseases and Risk Factors, 1990–2019: Update From the GBD 2019 Study. J. Am. Coll. Cardiol..

[2] van den Berg A., Dowdy S.F. (2011). Protein transduction domain delivery of therapeutic macromolecules. Curr. Opin. Biotechnol..

[3] Philippe G.J.B., Craik D.J., Henriques S.T. (2021). Converting peptides into drugs targeting intracellular protein-protein interactions. Drug Discov. Today.

[4] Petta I., Lievens S., Libert C., Tavernier J., De Bosscher K. (2016). Modulation of Protein-Protein Interactions for the Development of Novel Therapeutics. Mol. Ther..

[5] Tsomaia N. (2015). Peptide therapeutics: Targeting the undruggable space. Eur. J. Med. Chem..

[6] Muttenthaler M., King G.F., Adams D.J., Alewood P.F. (2021). Trends in peptide drug discovery. Nat. Rev. Drug Discov..

[7] Sabbah H.N. (2022). Elamipretide for Barth syndrome cardiomyopathy: Gradual rebuilding of a failed power grid. Heart Fail. Rev..

[8] Qvit N., Disatnik M.H., Sho E., Mochly-Rosen D. (2016). Selective Phosphorylation Inhibitor of Delta Protein Kinase C-Pyruvate Dehydrogenase Kinase Protein-Protein Interactions: Application for Myocardial Injury In Vivo. J. Am. Chem. Soc..

[9] Copolovici D.M., Langel K., Eriste E., Langel U. (2014). Cell-penetrating peptides: Design, synthesis, and applications. ACS Nano.

[10] Guidotti G., Brambilla L., Rossi D. (2017). Cell-Penetrating Peptides: From Basic Research to Clinics. Trends Pharmacol. Sci..

[11] Walensky L.D., Bird G.H. (2014). Hydrocarbon-stapled peptides: Principles, practice, and progress. J. Med. Chem..

[12] Verdine G.L., Hilinski G.J. (2012). Stapled peptides for intracellular drug targets. Methods Enzymol..

[13] Cromm P.M., Spiegel J., Grossmann T.N. (2015). Hydrocarbon stapled peptides as modulators of biological function. ACS Chem. Biol..

[14] Kumar D., Lisok A., Dahmane E., McCoy M., Shelake S., Chatterjee S., Allaj V., Sysa-Shah P., Wharram B., Lesniak W.G. (2019). Peptide-based PET quantifies target engagement of PD-L1 therapeutics. J. Clin. Investig..

[15] Kumar P., Tripathi S.K., Chen C.P., Wickstrom E., Thakur M.L. (2019). Evaluating Ga-68 Peptide Conjugates for Targeting VPAC Receptors: Stability and Pharmacokinetics. Mol. Imaging Biol..

[16] Notni J., Hermann P., Dregely I., Wester H.J. (2013). Convenient synthesis of ^68^Ga-labeled gadolinium(III) complexes: Towards bimodal responsive probes for functional imaging with PET/MRI. Chemistry.

[17] Notni J., Simecek J., Hermann P., Wester H.J. (2011). TRAP, a powerful and versatile framework for gallium-68 radiopharmaceuticals. Chemistry.

[18] Shoji-Kawata S., Sumpter R., Leveno M., Campbell G.R., Zou Z., Kinch L., Wilkins A.D., Sun Q., Pallauf K., MacDuff D. (2013). Identification of a candidate therapeutic autophagy-inducing peptide. Nature.

[19] Vives E., Brodin P., Lebleu B. (1997). A truncated HIV-1 Tat protein basic domain rapidly translocates through the plasma membrane and accumulates in the cell nucleus. J. Biol. Chem..

[20] Bulluck H., Yellon D.M., Hausenloy D.J. (2016). Reducing myocardial infarct size: Challenges and future opportunities. Heart.

[21] Sahoo S., Kariya T., Ishikawa K. (2021). Targeted delivery of therapeutic agents to the heart. Nat. Rev. Cardiol..

[22] Cicha I. (2021). The Grand Challenges in Cardiovascular Drug Delivery. Front. Drug Deliv..

[23] Feldman K.S., Pavlou M.P., Zahid M. (2021). Cardiac Targeting Peptide: From Identification to Validation to Mechanism of Transduction. Methods Mol. Biol..

[24] Spinelli T., Calcagnile S., Giuliano C., Rossi G., Lanzarotti C., Mair S., Stevens L., Nisbet I. (2014). Netupitant PET imaging and ADME studies in humans. J. Clin. Pharmacol..

[25] Im C., Kim H., Zaheer J., Kim J.Y., Lee Y.J., Kang C.M., Kim J.S. (2022). PET Tracing of Biodistribution for Orally Administered ^64^Cu-Labeled Polystyrene in Mice. J. Nucl. Med..

[26] Pohle K., Notni J., Bussemer J., Kessler H., Schwaiger M., Beer A.J. (2012). ^68^Ga-NODAGA-RGD is a suitable substitute for ^18^F-Galacto-RGD and can be produced with high specific activity in a cGMP/GRP compliant automated process. Nucl. Med. Biol..

[27] Tolmachev V., Stone-Elander S. (2010). Radiolabelled proteins for positron emission tomography: Pros and cons of labelling methods. Biochim. Biophys. Acta.

[28] Osborne B.E., Yue T.T.C., Waters E.C.T., Baark F., Southworth R., Long N.J. (2021). Synthesis and ex vivo biological evaluation of gallium-68 labelled NODAGA chelates assessing cardiac uptake and retention. Dalton. Trans..

[29] Katsila T., Siskos A.P., Tamvakopoulos C. (2012). Peptide and protein drugs: The study of their metabolism and catabolism by mass spectrometry. Mass Spectrom. Rev..

[30] Schwarze S.R., Ho A., Vocero-Akbani A., Dowdy S.F. (1999). In vivo protein transduction: Delivery of a biologically active protein into the mouse. Science.

[31] Stalmans S., Gevaert B., Wynendaele E., Nielandt J., De Tre G., Peremans K., Burvenich C., De Spiegeleer B. (2015). Classification of Peptides According to their Blood-Brain Barrier Influx. Protein Pept. Lett..

[32] Miyaji Y., Walter S., Chen L., Kurihara A., Ishizuka T., Saito M., Kawai K., Okazaki O. (2011). Distribution of KAI-9803, a novel delta-protein kinase C inhibitor, after intravenous administration to rats. Drug Metab. Dispos..

[33] Cardinale D., Colombo A., Bacchiani G., Tedeschi I., Meroni C.A., Veglia F., Civelli M., Lamantia G., Colombo N., Curigliano G. (2015). Early detection of anthracycline cardiotoxicity and improvement with heart failure therapy. Circulation.

[34] Zhang S., Liu X., Bawa-Khalfe T., Lu L.S., Lyu Y.L., Liu L.F., Yeh E.T. (2012). Identification of the molecular basis of doxorubicin-induced cardiotoxicity. Nat. Med..

[35] Russo M., Della Sala A., Tocchetti C.G., Porporato P.E., Ghigo A. (2021). Metabolic Aspects of Anthracycline Cardiotoxicity. Curr. Treat. Options Oncol..

[36] Alves A.C., Magarkar A., Horta M., Lima J., Bunker A., Nunes C., Reis S. (2017). Influence of doxorubicin on model cell membrane properties: Insights from in vitro and in silico studies. Sci. Rep..

[37] Baar M.P., Brandt R.M.C., Putavet D.A., Klein J.D.D., Derks K.W.J., Bourgeois B.R.M., Stryeck S., Rijksen Y., van Willigenburg H., Feijtel D.A. (2017). Targeted Apoptosis of Senescent Cells Restores Tissue Homeostasis in Response to Chemotoxicity and Aging. Cell.

[38] Nam S.H., Park J., Koo H. (2023). Recent advances in selective and targeted drug/gene delivery systems using cell-penetrating peptides. Arch. Pharm. Res..

[39] Xie J., Bi Y., Zhang H., Dong S., Teng L., Lee R.J., Yang Z. (2020). Cell-Penetrating Peptides in Diagnosis and Treatment of Human Diseases: From Preclinical Research to Clinical Application. Front. Pharmacol..

[40] Man F., Gawne P.J., de Rosales R.T. (2019). Nuclear imaging of liposomal drug delivery systems: A critical review of radiolabelling methods and applications in nanomedicine. Adv. Drug Deliv. Rev..

[41] Zou L., Peng Q., Wang P., Zhou B. (2017). Progress in Research and Application of HIV-1 TAT-Derived Cell-Penetrating Peptide. J. Membr. Biol..

[42] Ackers-Johnson M., Li P.Y., Holmes A.P., O’Brien S.M., Pavlovic D., Foo R.S. (2016). A Simplified, Langendorff-Free Method for Concomitant Isolation of Viable Cardiac Myocytes and Nonmyocytes From the Adult Mouse Heart. Circ. Res..

[43] Varasteh Z., Mohanta S., Li Y., Lopez Armbruster N., Braeuer M., Nekolla S.G., Habenicht A., Sager H.B., Raes G., Weber W. (2019). Targeting mannose receptor expression on macrophages in atherosclerotic plaques of apolipoprotein E-knockout mice using ^68^Ga-NOTA-anti-MMR nanobody: Non-invasive imaging of atherosclerotic plaques. EJNMMI Res..

[44] Staniszewska M., Fragoso Costa P., Eiber M., Klose J.M., Wosniack J., Reis H., Szarvas T., Hadaschik B., Luckerath K., Herrmann K. (2021). Enzalutamide Enhances PSMA Expression of PSMA-Low Prostate Cancer. Int. J. Mol. Sci..

[45] Bloom M.W., Hamo C.E., Cardinale D., Ky B., Nohria A., Baer L., Skopicki H., Lenihan D.J., Gheorghiade M., Lyon A.R. (2016). Cancer Therapy-Related Cardiac Dysfunction and Heart Failure: Part 1: Definitions, Pathophysiology, Risk Factors, and Imaging. Circ. Heart Fail..

[46] Bauckneht M., Ferrarazzo G., Fiz F., Morbelli S., Sarocchi M., Pastorino F., Ghidella A., Pomposelli E., Miglino M., Ameri P. (2017). Doxorubicin Effect on Myocardial Metabolism as a Prerequisite for Subsequent Development of Cardiac Toxicity: A Translational ^18^F-FDG PET/CT Observation. J. Nucl. Med..

[47] Bauckneht M., Pastorino F., Castellani P., Cossu V., Orengo A.M., Piccioli P., Emionite L., Capitanio S., Yosifov N., Bruno S. (2020). Increased myocardial ^18^F-FDG uptake as a marker of Doxorubicin-induced oxidative stress. J. Nucl. Cardiol..

