# Supplementary figures and images for: Demonstration of the Early Cardiac Bioavailability of a Non-Specific Cell-Targeted Peptide Using Radionuclide-Based Imaging In Vivo
Source: Pharmaceuticals (Basel). 2023 May 31;16(6):824. doi: 10.3390/ph16060824 (PMC10300709; doi:10.3390/ph16060824)

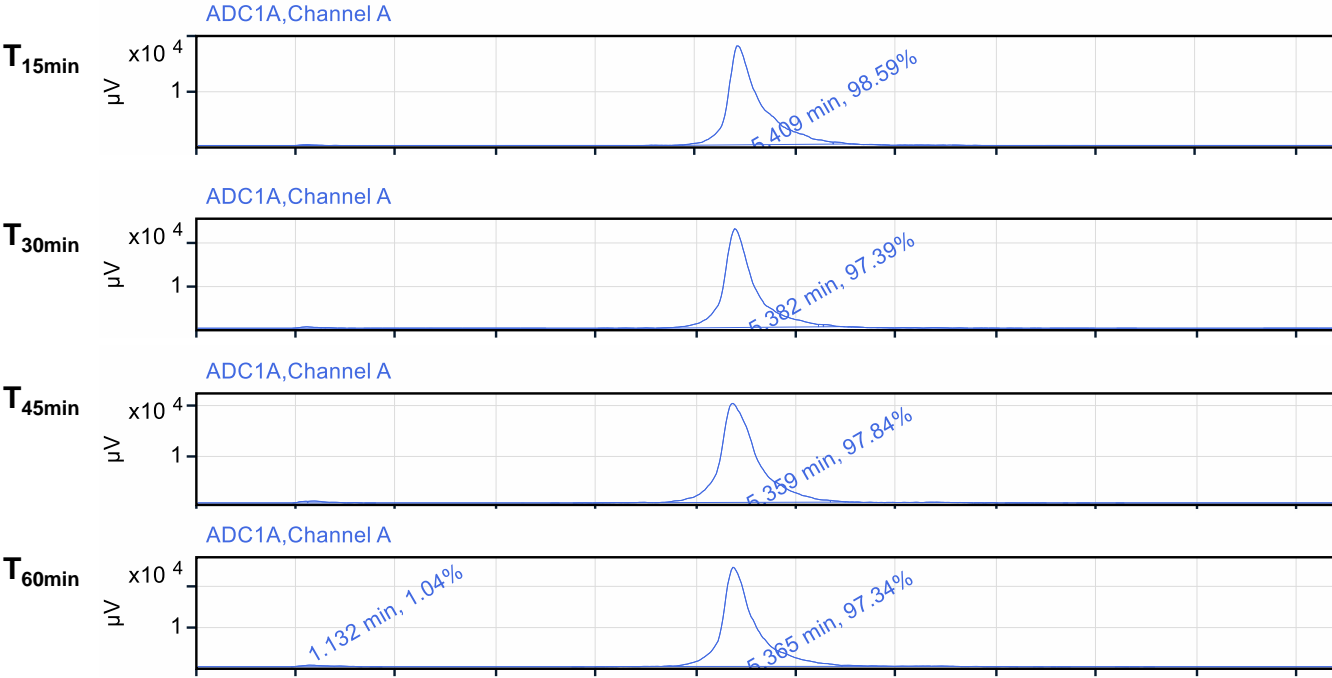

FIG S1

**a** Cardiac lysate

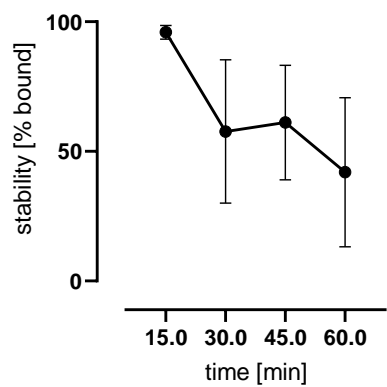

**b**

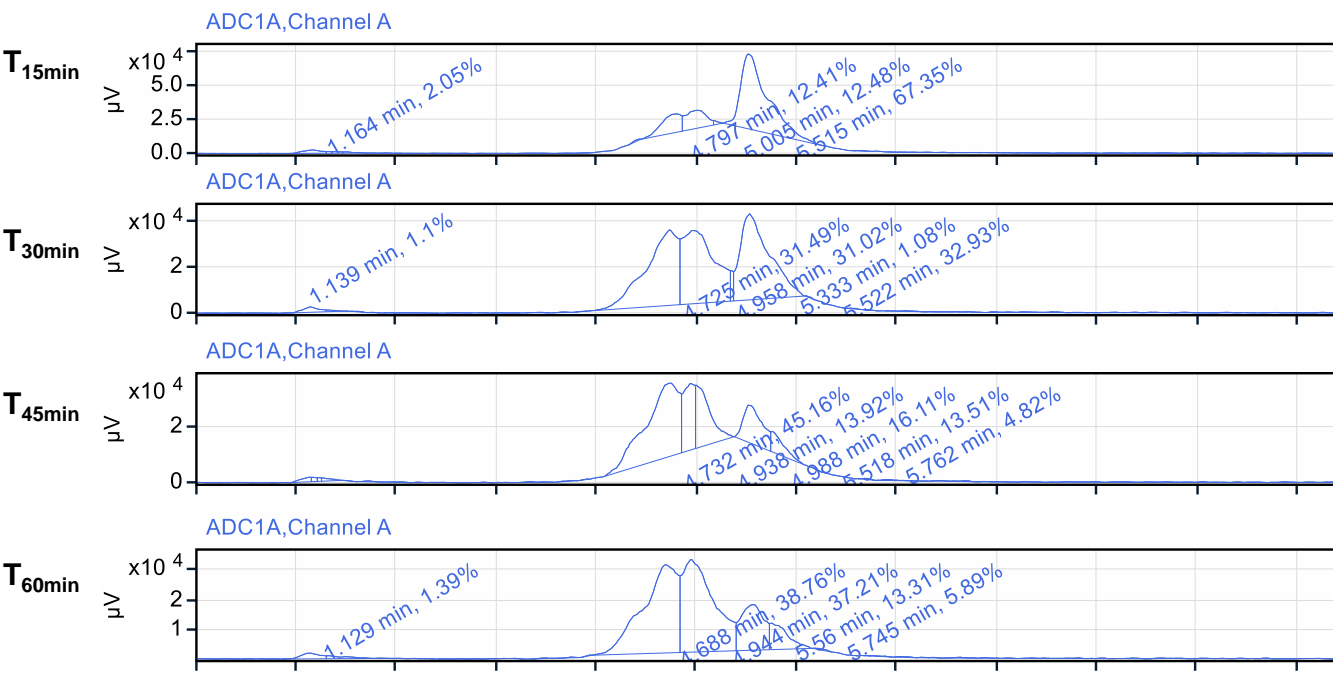

FIG S2

**a**  
Proteinase K

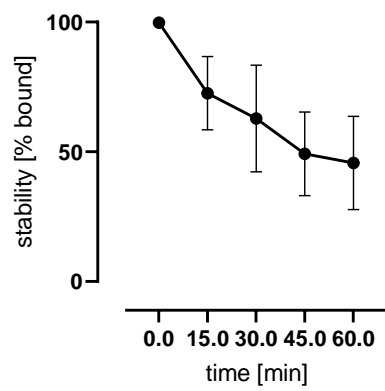

**b**

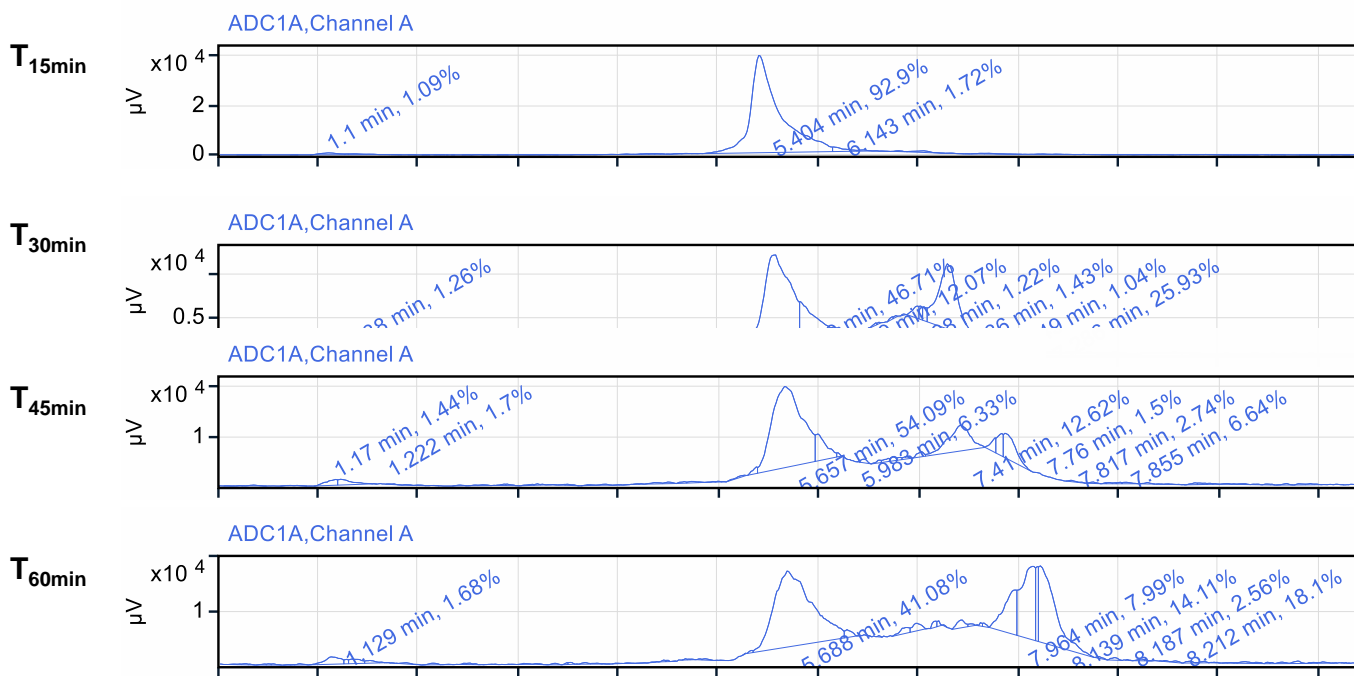

FIG S3

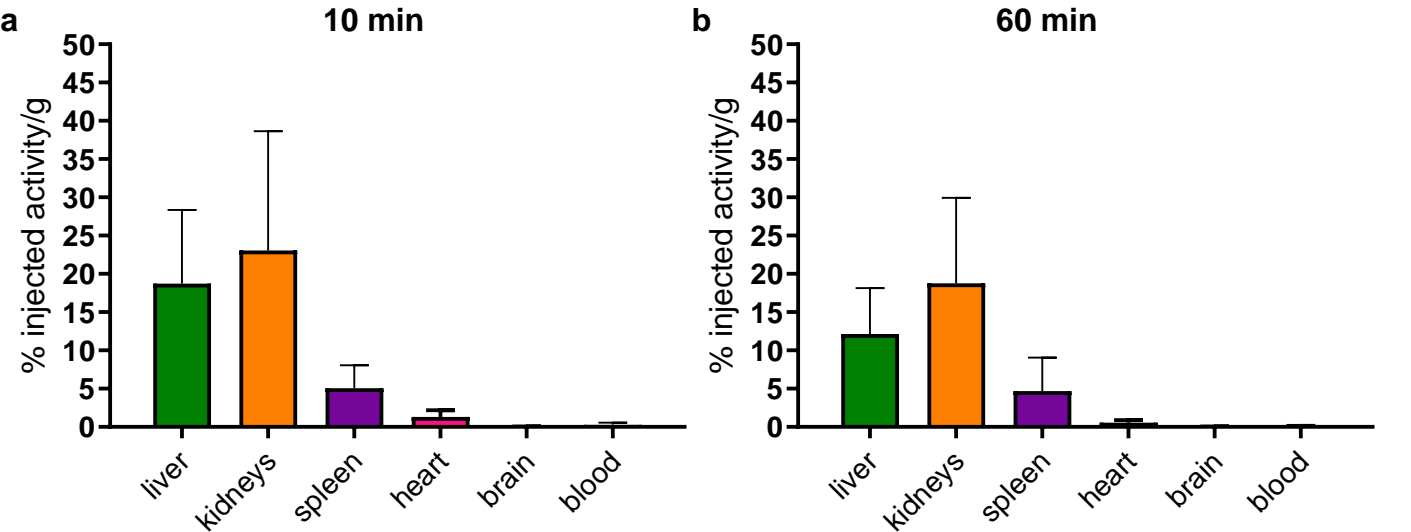

FIG S4

Supplement: Supplementary file 1 [file pharmaceuticals-16-00824-s001.zip › pharmaceuticals-2343103-supplementary.pdf]
